# Bridging the gap between academia and practice: novel organogram at the Pharmacy Council of India

**DOI:** 10.1186/s40545-022-00416-0

**Published:** 2022-03-20

**Authors:** Mansi Doshi, Minesh Parbat, Vibhu Paudyal, John Marriott

**Affiliations:** 1 Medicines Optimisation Support and Services Development, Mumbai & Vadodara, India; 2 School of Pharmacy, University of Birmingham, Birmingham, B15 2TT UK

**Keywords:** Novel organogram, Pharmacy Council of India, Clinical Pharmacists’ Registrations, Licenses, Registration examinations, Registration renewal requirements

## Abstract

Harm with inappropriate and irrational use of medications is a global challenge. The need for and patient access to medicines optimisation services is apparent globally and amplified in India due to multiple reasons. Clinical pharmacists are ideally placed to promote patient safety with medicines use optimisation and other pharmaceutical care services through appropriate legislative, policy, and compensation mechanisms to achieve optimal patient outcomes. The need is for a move at a global level, an enabling organisational structure at Pharmacy Councils and in practice regulations, particularly in countries where clinical roles are still in infancy. This narrative describes the current status and future needs for development of medicines optimisation services across sectors through regulatory and organisational reforms at the Pharmacy Council of India with additional registration, continuing professional development, renewal and licensing requirements for clinical pharmacists to respond to patient and societal needs in India.

## Introduction

The WHO Global Patient Safety Challenge 2017 [1] aims to decrease harm with use of medicines. Pharmacists’ role and contribution to medicines optimisation, “the safe and effective use of medicines with the right choice, at the right time, and with patients being engaged in the process by their clinical team to enable best possible outcomes” [2, 3] is being increasingly recognized.

The need for and patient access to medicines optimisation services is apparent. This need is amplified in India where patient involvement in their prescriber-led therapeutic management is minimal. In addition, access to and self-medication of prescription and non-prescription medications is a common occurrence. Globally, challenges of health care delivery, including preventive or supportive care, access, safety, quality and cost have been highlighted in the past [4]. Clinical pharmacists are ideally placed to promote patient safety with medicines use optimisation and other pharmaceutical care services through appropriate legislative, policy, and compensation mechanisms to achieve optimal patient outcomes. The need is for a move at a global level, an enabling organisational structure at Pharmacy Councils and in practice regulations, particularly in countries where clinical roles are still in infancy. FIP developmental goals (DGs) on policy development and reform (DG 13) [5], access to medicines, expertise, appropriate medicines information and services towards patient safety with medicines use optimisation (DG 14, 15, 18, 19) [5] lay the foundation to such reforms required globally. This narrative will aim to describe the current status and future needs for development of medicines optimisation services across sectors through regulatory and organisational reforms at the Pharmacy Council of India (PCI) with additional registration, continuing professional development (CPD), renewal and licensing requirements for clinical pharmacists to respond to patient and societal needs in India, in line with FIP DG 13 [5].

### Evidence base for clinical pharmacists roles’

Benefits and challenges of integrating pharmacists in non-dispensing roles in primary care, with distribution by sector in selected countries is reported by Komwong and co-workers [6]. Systematic reviews have also demonstrated that evolution of pharmacists’ non-dispensing patient-facing and targeted clinical role beyond medicines supply have improved access to primary care services. Beneficial impact has been demonstrated on health system indicators and in patients with chronic diseases [7, 8] with small improvements in clinical outcomes, quality of life, and reduction of health service utilization, with medicines use education and counselling by pharmacists at different points of care [9]. Positive effects of integration of clinical pharmacists into primary care general practice clinics on primary outcomes related to medication use or clinical outcomes have also been reported in 19 of the 38 studies included in another review [10]. Meta-analyses of 17 studies with common endpoints such as blood pressure, glycosylated haemoglobin, cholesterol and/or Framingham risk score, favour pharmacist intervention, with significant improvements in clinical outcomes in intervention patients compared to control patients [10]. A small but prospective before-after intervention study (Pharmacists in Practice Study, that is, PIPS) in primary care clinics has also reported a significant decrease in medication related problems with significant positive impact on adherence to medications and patient satisfaction with pharmacists’ consultation [11]. Thus, collaborative work as part of the health care team may, in all settings including primary care, improve overall patient care and health outcomes. A retrospective study evaluating clinical pharmacists’ activity in general practice has also reported significant financial returns on investment with efficient delivery of clinical interventions in high volume [12]. Extrapolating this to countries where patients pay for health care, development of clinical pharmacy services in primary care may decrease cost to the patient and improve access to good pharmaceutical care across sectors, medication adherence and patient outcomes.

Evidence clearly highlights that access to evidence-based information with medicines use reviews, interventions, education, counselling and optimisation per se may support patients and the healthcare team and system [2, 3, 13–23].

### Development of clinical pharmacy services in primary care in India

In India, clinical pharmacy and medicines optimisation services exist in some hospitals. In addition, although slow paced, these services and their uptake are on the rise. However, these are virtually non-existent in primary care. In 2011, Shah [24] developed in Western India, a practice in community with a registered medical practitioner, with the aim of improving access to clinical pharmacy services. Lack of willingness of doctors and management in hospitals and community pharmacies towards development of consultation areas and clinical pharmacist-led medicines optimisation clinics (MOCs) led to pioneering an independent model of community practise with direct access and referral-based services, incorporating essential, advanced and enhanced services, and a remote clinic [24]. Establishing supplementary and independent prescriber status or widespread patient-focussed clinical pharmacy services necessary to deliver health agendas in India are long-term aspirations. However, implementation of a blend of in-community, hospital and virtual clinics, in-patient and healthcare interface services with application of artificial intelligence and digitalisation could enhance patient care.

### Clinical pharmacy/pharmacy practice education qualifications, Pharmacy Practice Regulations & PCI organogram

India offers multiple options in pharmacy education. But graduation and registration do not make pharmacists with different qualifications practice ready. This is due to lack of patient and practice focus at the undergraduate BPharm degree and diploma in pharmacy. Clinical pharmacy/pharmacy practice was first introduced in academia in India as a master’s degree in 1996 [25] followed by the PharmD in 2008 [26]. The lag in Pharmacy Practice Regulations (PPR) that followed only in 2015 [27] led to challenges. Thus, it is essential to outline here this timeline (Table 1).

**Table 1:** Timeline and lag between introduction of pharmacy practice regulations and clinical pharmacy degrees

It is Over…
5 years since the Pharmacy Practice Regulations 2015
6 years since the first PharmD graduates
12 years since the first PharmD admissions
20 years since the first in-country clinical pharmacy/pharmacy practice graduates (2-year masters’ degree post BPharm)
Recent developments
As a first, 2019 Amendment [28] to PPR 2015 [27]-mention of Clinical Pharmacists (hospital centric practice and PharmD graduates only, and with no detail on responsibilities) and Drug Information Centres in hospitals
2021 Amendment [29] to PPR 2015 [27]-inclusion of roles and responsibilities of PCI’s PharmD Clinical Pharmacists in hospitals (as appropriate) and addition of Drug Information Pharmacists in Hospitals (in addition to “Details of Position Title and job responsibilities of Drug Information Pharmacist at Pharmacy practice site in a health care setting (Drug store/Pharmacy)” in Appendix III of PPR 2015 [27]

**Fig. 1:**
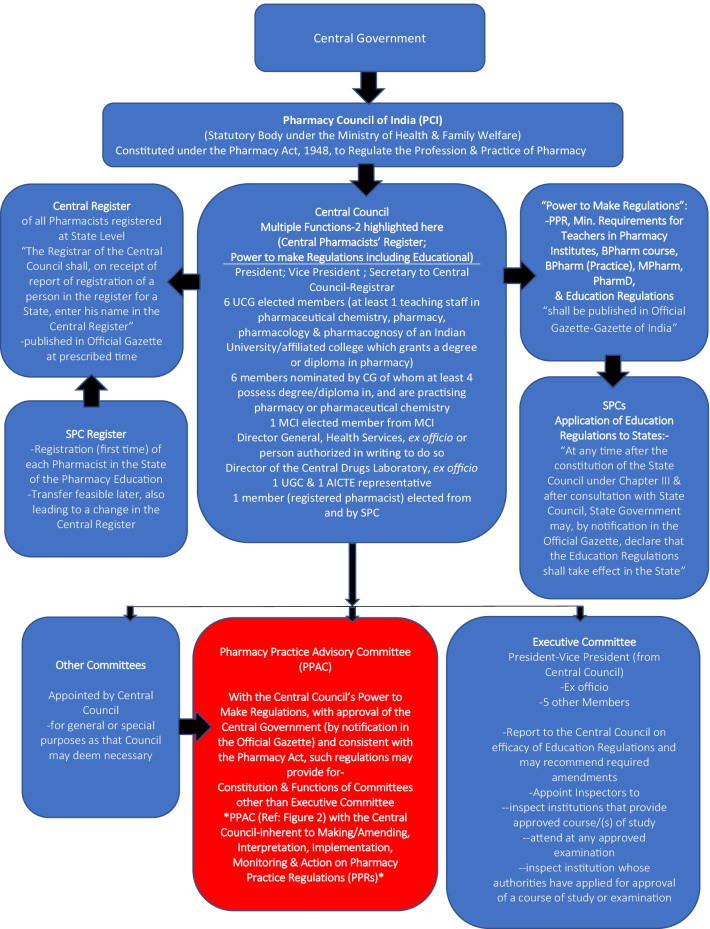
Pharmacy Council of India, Registers and Committees [32]

Currently, with about 270 colleges in India offering the PharmD degree [30] in addition to the master’s in clinical pharmacy/pharmacy practice, clinical pharmacists’ numbers are rising exponentially. But, the lag in introduction of practice regulations [27, 31] and void in planning and early on inclusive organisational structure at the PCI (Fig. 1) for clinical pharmacy practice and professionals had led to open-ended roles, responsibilities and PharmD graduates’ led community-based pharma clinics. This led the PCI in its 2015 clarification [31] to not permit pharma clinics to ‘diagnose disease and prescribe medicines’, a likely result of unanticipated practice challenges with the PharmD graduates’ title of ‘Dr’ causing a dilemma. But, a lack of direct patient care opportunities for a significant number of the 8,000 + PharmD graduates annually led to protests and inclusion of “PCI’s” PharmD clinical pharmacists in the 2019 amendment [28] to PPR 2015 to optimise therapy but ‘only hospital-centric and only in association with physicians or other health professionals, recognising only PharmD graduates as clinical pharmacists and belatedly, for the first time, in 2021 [29], outlining roles and responsibilities in hospitals (as appropriate)’.

### Proposed add-on organogram at PCI: registration and regulatory amendments

**Fig. 2:**
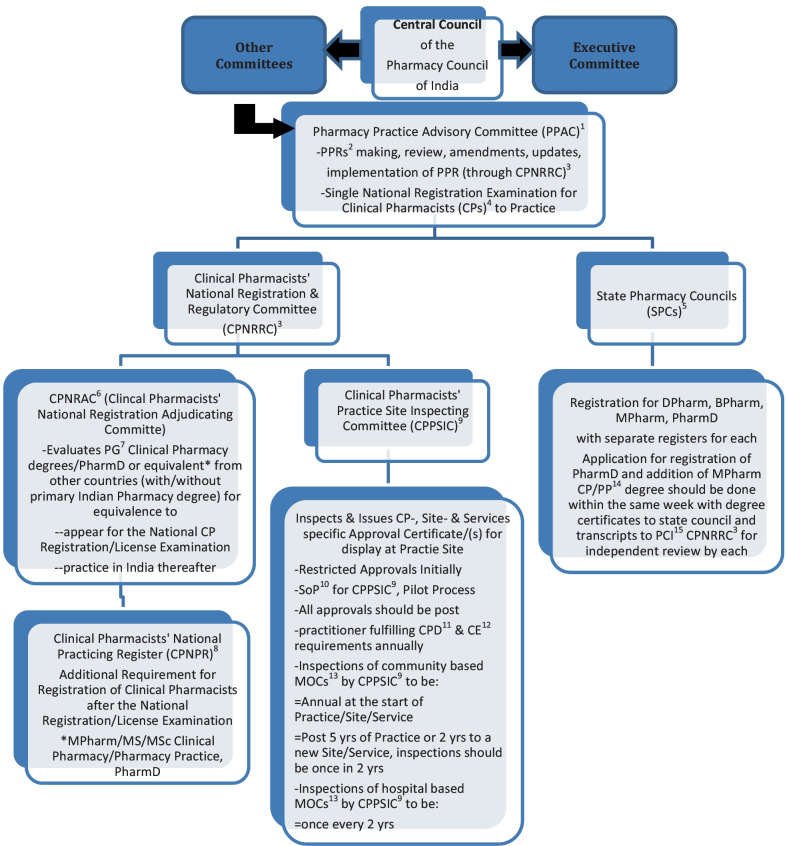
Vision of a Novel Add-On Organogram at the Pharmacy Council of India. ^1^Pharmacy Practice Advisory Committee. ^2^Pharmacy Practice Regulations. ^3^Clinical Pharmacists’ National Registration and Regulatory Committee. ^4^Clinical Pharmacists. ^5^State Pharmacy Councils. ^6^ Clinical Pharmacists’ National Registration Adjudicating Committee. ^7^Post graduate. ^8^Clinical Pharmacists’ National Practicing Register. ^9^Clinical Pharmacists’ Practice Site Inspecting Committee. ^10^Standard Operating Procedure. ^11^Continuing Professional Development. ^12^Continuing Education. ^13^Medicines Optimisation Clinics. ^14^Pharmacy practice. ^15^Pharmacy Council of India

The Pharmacy Act, 1948 [32] under which PCI is constituted to regulate the profession and practice of pharmacy, requires state wise registrations for all pharmacists (diploma, degree, practising, non-practising) (Figs. 1, 2). But a lack of clarity on registers for variably qualified practising and non-practising pharmacists, inadequate registration, CPD, renewal and clinical practice license requirements demands urgent and imminent change.

Statutory and regulatory reforms, and a novel framework may strengthen existing policies, regulations and enforceable legislation for continuous pharmaceutical workforce and practice development. It will also extend the scope of pharmacy practice and service development in line with FIP DG 13 [5]. This in turn will promote responsible use of medicines by mobilising medicines expertise and implement high-value cognitive services to review and optimize medicines use (FIP DG 14) [5].

A model add-on (Fig. 2) to the organisational structure at the PCI (Fig. 1) may be the first step with a ‘Pharmacy Practice Advisory Committee’ (PPAC) (Figs. 1, 2) and subcommittees under the PCI’s Central Council. Regulatory transparency of Councils’ ongoing activities, plans and procedures, intervals and timeframes, and open consultations with pharmacists in practice, research and academia may encourage and enhance development and improve accountability and acceptability of professionals.

The proposed organogram will strengthen the scope and prospects for the ‘future pharmacist’ in medicines optimisation [20–23, 33] across sectors. For India, the framework proposed in this manuscript highlights streamlining clinical pharmacists’ degrees, registrations and amendments to include:

#### Proposed responsibilities within the proposed organogram

Proposed responsibilities of the Pharmacy Practice Advisory Committee and its subcommittees are highlighted in Fig. 2. The Clinical Pharmacists’ National Registration and Regulatory Committee (CPNRRC), under the Pharmacy Practice Advisory Committee, is responsible for registration of clinical pharmacists, maintaining a Clinical Pharmacists’ National Practicing Register (CPNPR), review and updates, at defined regular intervals, of Pharmacy Practice Regulations applicability, interpretation and implementation for practising clinical pharmacists across sectors, and a Clinical Pharmacists’ Practice Site Inspecting Committee (CPPSIC-referred to hereafter as Inspecting Committee) under its umbrella.

#### Proposed pharmacists’ registers

There is clear need for practising (national for clinical pharmacists, and state wise) and non-practising (state wise only) registers. Among the state practising registers, 3 registers may be maintained separately for clinical pharmacists, BPharm and *other* MPharm graduates who aim to or are practising out of dispensing pharmacies in hospitals/community, and for graduates with diploma in pharmacy (2 years course after A levels)-as pharmacy technicians.

#### Proposed registration examinations, registrations and renewals

All pharmacists, including clinical pharmacists are required to register with respective the State Pharmacy Council (SPC). Clinical pharmacists in practice and clinical pharmacy preceptors/teacher-practitioners involved in training and supervising pharmacy students in clinical settings and with patient-facing scenarios also require additional registration with the Clinical Pharmacists’ National Practicing Register. Only clinical pharmacists who choose to work solely in academia or industry without direct patient facing responsibilities may be exempt.

The proposed Pharmacy Practice Advisory Committee will be responsible for planning and conducting a *national registration/license examination* after the clinical pharmacy degree (MPharm/MS/MSc Clinical Pharmacy/Pharmacy Practice/PharmD that includes a 12-month internship) for all clinical pharmacists who aim to be in practice. This would lead to inclusion on the Clinical Pharmacists’ National Practicing Register in addition to State wise registration. This will allow practice across sectors and states.

*The existing State Pharmacy Councils* conduct a similar proposed *examination at state level*-for all degree pharmacists and a separate one for diploma (DPharm) pharmacists leading to pharmacy technician responsibilities and role for the latter.

Pharmacists’ registrations at national and state levels are to be used in parallel in case of legal or disciplinary action. State Pharmacy Councils may be rightly placed to take on the responsibility to review and restrict inappropriate behaviour and pharmacy practice, in association with a litigation committee under the CPNRRC and overseen by the Pharmacy Practice Advisory Committee.

#### Proposed inspections

This Inspecting Committee is responsible for inspection (details on frequency: Fig. 2) and regulation of clinical pharmacy services, medicines optimisation clinics including processes and procedures, in hospitals and community and random review of recordings from virtual or remote clinics.

The CPPSIC approval certificates/licenses are clinical pharmacist-, site/(s) and services (categorised checkboxes) specific, and to be displayed at practice site with clear indication of license to practice with a registered medical practitioner or for independent practice. For virtual and remote clinics, there may be PCI and Inspecting Committee portal that would directly flag up a digital license during each virtual/remote consultation, to be conducted through a PCI approved software. But these clinical pharmacy and medicines optimisation services and practice sites in primary care demand development of Ministry of Health and Family Welfare (MoH&FW) approved over-the-counter-, pharmacy- and prescription only medicine lists/formulary. An additional non-medical (clinical pharmacist) prescribing list may become relevant with further reforms in the practice regulations permitting prescribing by clinical pharmacists with additional training and accreditation.

#### Proposed registration renewals requirements

Registration renewals should be at state and national levels. For clinical pharmacists, renewals on CPNPR may be annual, with statewise renewals every 2 years. For independent private practice, clinical pharmacists’ practice site information will be required to be included in the practising register. For all other pharmacists (BPharm, MPharm) registered with the SPC and in practice, renewals would be every year with the SPC.

Pharmacists CPD requirement should be more than state level update programmes existing at different State Pharmacy Councils in India, or attending a ‘major/national pharmacy meeting’ [20], and in line with those proposed in Table 2.

**Table 2:** Proposed pharmacists’ registration renewal requirements

1	Fitness to practice declaration
2	Clinical Pharmacists National Registration Renewals Requirement
2.1	Specified number and type of CPD records (with at least one each starting at each stage of the CPD cycle, and at least one peer review discussion-based learning) and accredited CE and recorded CPD requirement to be submitted (digitally on a CPD portal of the Council, 3 months prior to renewal) to, and reviewed by the responsible Pharmacy Council
2.2	8 CPD records to be submitted to for review by the ‘Clinical Pharmacists’ National Registration Adjudicating Committee’ (CPNRAC)
2.3	2 accredited continuing education (CE) (2) activities to be undertaken and accreditation document submitted to CPNRAC
2.3	No additional CPD submission requirements to the SPC every year for registration renewal
3	All other pharmacists in practice
3.1	Specific CPD and CE requirements on similar lines as outlined for clinical pharmacists, with CPD records and accredited CE documents submission to SPC for review and approval
4	Diploma Pharmacists
4.1	To be identified as pharmacy technicians
4.2	CPD and CE Requirements to be laid down uniformly by SPC across states
5	Every pharmacist is required to declare that all CPD records are his/her own If otherwise and in case of plagiarism, pharmacist registration will be at stake as inappropriate behaviour and action at any time in professional practice

CPD activities and records submitted for review are required to demonstrate significant learning and upskilling to improve service provision and practice, and enable the pharmacist to better support the patient in their therapeutic management and health outcomes.

### Patient-focused training and professional development in education

Essential to this proposed framework in India is also to incorporate clinical pharmacy and practice as a compulsory subject early on in the undergraduate pharmacy degree with adequate patient and practice-focus in teaching and training. CPD should be an integral part of the pharmacy professional degree and introduced in the first year of education and built on gradually. The need is to develop uniform CPD requirements and guidance for pharmacists’ registrations/licenses, renewals and continuation of practice, and a digital CPD platform for individual pharmacists and national pharmacy councils. This will lead the future pharmacist to be a competent, skilled global pharmacist.

In March 2020, the Supreme court of India ruled PCI as the sole regulator of Pharmacy education in India [34] in addition to the existing regulatory role. This presents significant autonomy and opportunity to the PCI to implement change. The COVID-19 pandemic led the government in India to recognise pharmacists as essential health workforce. Time cannot be more apt to work with the MoH&FW, towards these reforms with development of national and local clinical guidelines under the purview of a proposed ‘*National Clinical Evidence-Based Review committee’*. With select representation of clinical experts and nominated members of the Indian Medical Association, National Medical Council, Indian Pharmaceutical Association, Pharmacy Council of India and Indian Nursing Council, open consultation with councils, practitioners across states, and central and state governments will lead to national guidelines for management of different disorders/diseases, followed on by appropriate implementation and may lead to local guidelines based on health scenarios. These guidelines may be accessible through Specialist National Organisation portals or websites, or National Programmes/WHO/relevant others.

This amalgamated with development of clinical pharmacy services and medicines optimisation clinics across sectors and at health interfaces may present adequate avenues in the social movement of the WHO patient safety challenge towards safer and better health outcomes. With a target to reduce harm with medicines by 50% by 2022 [1] and the inherent challenges of the pandemic, it is never too late for clinical pharmacists to join the existing movement. But, as suggested by Giberson and co-workers [4], an opportunity exists for health leadership and policy makers to support and implement additional, existing and evidence‐based models of cost‐effective pharmacist‐delivered patient care.

## Conclusion

The common goal of improved and safer healthcare through medicines optimisation, should recognise the contribution of clinical pharmacists in achieving seamless care and better health outcomes. Regulatory reforms, early training, and CPD, implementation of the proposed organogram, regulatory support with inclusiveness and inherent transparency, consultations and medicines optimisation services across sectors and at interface, is expected to lead to positive contribution of pharmacists in achieving patient safety challenges in the future.

## Data Availability

Not applicable.

## Abbreviations

WHO: World Health Organisation
FIP: International Pharmaceutical Federation
DG: Developmental Goals
PCI: Pharmacy Council of India
CPD: Continuing Professional Development
PIPS: Pharmacists in Practice Study
MOC: Medicines Optimisation Clinic
BPharm: Bachelor of Pharmacy
PharmD: Doctor of Pharmacy
PPR: Pharmacy Practice Regulations
UGC: University Grants Commission
CG: Central Government
MCI: Medical Council of India
AICTE: All India Council for Technical Education
SPC: State Pharmacy Council
PPAC: Pharmacy Practice Advisory Committee
CPNRRC: Clinical Pharmacists’ National Registration and Regulatory Committee
CP: Clinical Pharmacist
CPNRAC: Clinical Pharmacists’ National Registration Adjudicating Committee
PG: Postgraduate
CPNPR: Clinical Pharmacists’ National Practicing Register
CPPSIC: Clinical Pharmacists’ Practice Site Inspecting Committee
SOP: Standard operating procedure
CE: Continuing education
PP: Pharmacy practice
DPharm: Diploma in Pharmacy
MoH&FW: Ministry of Health and Family Welfare
